# Protocol-based CT-guided brachytherapy for patients with prostate cancer and previous rectal extirpation—a curative approach

**DOI:** 10.1007/s00066-024-02266-y

**Published:** 2024-08-02

**Authors:** Philipp Schubert, Vratislav Strnad, Thomas Weißmann, Claudia Schweizer, Michael Lotter, Stephan Kreppner, Andre Karius, Rainer Fietkau, Ricarda Merten

**Affiliations:** 1 Department of Radiation Oncology, University Hospital Erlangen, Friedrich-Alexander-Universität Erlangen-Nürnberg, Universitätsstraße 27, 91054 Erlangen, Germany; 2 Comprehensive Cancer Center Erlangen-EMN, Erlangen, Germany

**Keywords:** CT-guided brachytherapy, Prostate cancer treatment, Rectal extirpation, Curative brachytherapy protocol, Prostate cancer brachytherapy, Radiation therapy prostate cancer, Image-guided brachytherapy, High-dose rate brachytherapy prostate

## Abstract

**Objective:**

There are numerous curative treatment possibilities for prostate cancer. In patients who have undergone rectal extirpation for rectal cancer treatment, curative options are limited due to anatomic changes and previous irradiation of the pelvis. In this analysis, we validate the feasibility of CT-guided transperineal interstitial brachytherapy for this specific scenario.

**Patients and methods:**

We analyzed the treatment procedures and outcomes of 5 patients with metachronic nonmetastatic prostate cancer. Ultrasound-guided brachytherapy was not possible in any of the patients. Of these 5 patients, 3 were treated for prostate cancer using temporary brachytherapy with Ir-192 only, and 2 were treated with external-beam radiation therapy and temporary brachytherapy as a boost. CT-guided brachytherapy was performed in all patients. We analyzed the feasibility, efficacy, treatment-related toxicity, and quality of life (EORTC-30, IEFF, IPSS, and ICIQ questionnaires) of the treatments.

**Results:**

Median follow-up was 35 months. Two out of five patients received boost irradiation (HDR 2 × 9 Gy, PDR 30 Gy). Three out of five patients were treated with PDR brachytherapy in two sessions up to a total dose of 60 Gy. Dosimetric parameters were documented as median values as follows: V100 94.7% (94.5–98.4%), D2_bladder_ 64.3% (50.9–78.3%), D10_urethra_ 131.05% (123.2%–141.2%), and D30_urethra_ 122.45% (116.2%–129.5%). At the time of analysis, no biochemical recurrence had been documented. Furthermore, neither early nor late side effects exceeding CTCAE grade 2 were documented.

**Conclusion:**

CT-guided transperineal brachytherapy of the prostate in patients with previous rectal surgery and radiation therapy is safe and represents a possible curative treatment option. Brachytherapy can be considered for patients with metachronic prostate cancer in this specific scenario, albeit preferably in experienced high-volume centers.

## Introduction

Advancements in rectal cancer treatment have led to excellent long-term survival rates. However, this effectiveness also seems to increase the risk of secondary malignancies, notably of prostate cancer, in patients treated for early rectal cancer [1]. Patients previously treated for rectal cancer, especially those who have undergone radiotherapy and rectal resection, present with unique challenges that require careful consideration when planning curative treatments for secondary malignancies like prostate cancer.

For nonmetastatic prostate cancer, the primary curative treatment options are radical prostatectomy and definitive radiation therapy, both of which have been shown to yield comparable outcomes [2]. Prostate cancer requires higher doses of radiation for effective tumor control as compared to other cancers [3]. This poses a challenge in patients who have previously received pelvic irradiation for rectal cancer, as the high dose levels necessary for prostate cancer cannot be achieved with external-beam radiation therapy (EBRT) without risking serious complications due to the already maxed-out tissue tolerance [4]. In such scenarios, brachytherapy emerges as the sole treatment modality capable of delivering the required high doses to the prostate.

Brachytherapy typically involves some form of image guidance, as it often uses iterative approaches that require the targeted organ to be accessed with needles or catheters for the radiation source. MRI and ultrasound are the gold standards in prostate cancer diagnosis, offering reliable imaging of both the target area and its spatial relationship to surrounding organs at risk. The region of interest is readily accessible via a transrectal ultrasound probe, making this the preferred method for image guidance in prostate brachytherapy. However, for patients who have had undergone rectal extirpation or significant rectal resection resulting in rectal lumen stenosis, transrectal ultrasound may not be feasible, thus complicating the use of ultrasound-based image-guided brachytherapy.

This report aims to explore the viability of radiation therapy for nonmetastatic prostate cancer in patients previously treated for rectal cancer with surgery and chemoradiation, particularly those who are anatomically ineligible for transrectal ultrasound-guided brachytherapy.

## Materials and methods

### Patients

This analysis included a total of 5 patients diagnosed with prostate cancer who were deemed eligible for definitive curative treatment according to national guidelines. Among them, 4 patients had undergone rectal extirpation along with additional chemoradiation, and 1 patient had undergone rectal resection with additional chemotherapy, thus rendering all participants unsuitable for transrectal ultrasound. The characteristics of the patients are detailed in Table 1. Prior to treatment, all patients underwent PSMA-PET-CT scans and MRI scans, which were taken into account during the implantation and treatment planning stages.

**Table 1:** Patient characteristics

Patient no.	1	2	3	4	5
Age at BT (years)	65	80	61	76	68
Gleason score	4 + 5 = 9	4 + 5 = 9	4 + 4 = 8	3 + 3 = 6	4 + 3 = 7 b
iPSA (ng/ml)	9.55	14.00	9.80	–	5.74
Prostate volume (ccm)	20.00	36.06	23.10	–	28.99
Pretreatment	Extirpation (rectal cancer)	Extirpation + RT (rectal cancer)	Extirpation + RT (rectal cancer)	Extirpation + RT (rectal cancer) + seed implantation (prostate-cancer)	nRCT + resection
Pretreatment EBRT (Gy)	50.4 (1.8)^a^	50.4 (1.8)/45 (1.8)^a^	50.4 (1.8)	40 (2)	50.4 (1.8)
MRI staging	cT2	cT2	–	cT2	cT2

*BT* Brachytherapy, *iPSA* initial Prostate Specific Antigen, *EBRT* External Beam radiotherapy, *MRI* magnet resonance imaging, *RT* Radiotherapy, *nRCT* neoadjuvant radio chemo therapy

^a^Patient received 45/50.4 Gy to the pelvic area upfront of brachytherapy.

Each patient provided written informed consent, which included a clause for data privacy regarding the collection and analysis of their information for research purposes.

Data were gathered from electronic patient records through a review of historical records.

### External-beam radiotherapy

Two patients received 45–50.4 Gy (1.8 Gy in 25–28 fractions) to the whole pelvis upfront of brachytherapy. One patient was radiotherapy naive, while the other qualified for whole-pelvis radiotherapy due to the high-risk nature of the disease. Additionally, given the long interval since the pretreatment, we decided to offer whole-pelvis radiotherapy as reirradiation. CTV/PTV delineation was according to NRG consensus guidelines [5]. Dose prescription was defined according to ICRU report 83. Volumetric modulated arc therapy was applied in both patients as EBRT with 6‑MeV photons.

### Brachytherapy

Two patients (2/5) underwent brachytherapy as a boost following EBRT for prostate cancer treatment. For those receiving brachytherapy as their sole treatment (3/5), two sessions of brachytherapy were scheduled with an interval of 3 weeks.

**Fig. 1:**
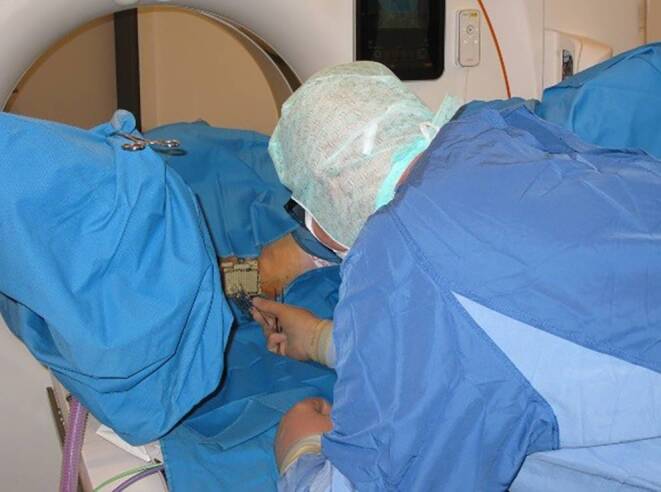
Implantation on the CT couch in lithotomy position

**Fig. 2:**
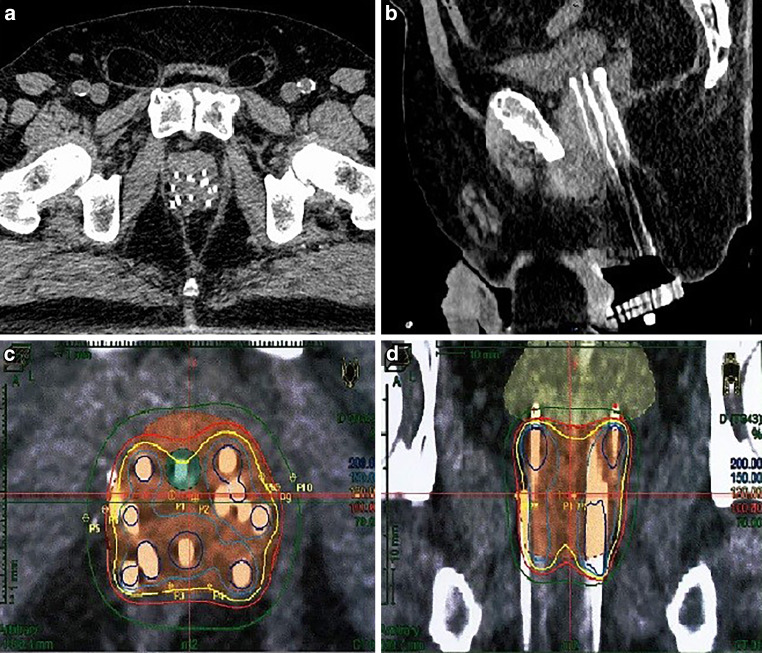
Example of CT-guided salvage brachytherapy in a patient after rectal extirpation; **a** axial view, **b** sagittal view, **c**, **d** corresponding dose distribution

The procedure for inserting needles was carried out under general anesthesia. Leg holders were installed on the CT couch to put the patient in lithotomy position. A bladder catheter was inserted. To minimize imaging artifacts, hollow 200-mm titanium needles were used. The first needle was implanted freehand under continuous CT guidance (Fig. 1), acting as a guide for subsequent needle placement. Thereafter, a template was used to ensure symmetrical placement of the following needles. With ongoing CT guidance, sufficient titanium needles were inserted into all relevant areas of the prostate, taking care to avoid nearby organs at risk. Once implantation was complete, the template was secured to the skin. The patient was then awakened from general anesthesia and moved to the brachytherapy treatment room, where treatment was delivered using a microSelectron v3 device (Nucletron B. V., Veenendaal, the Netherlands) equipped with a 192-Ir source. For boost irradiation, one patient (1/5) received high-dose-rate (HDR) brachytherapy with 2 × 9 Gy (43.2 Gy EQD2, α/β = 3 Gy) and one patient (1/5) received PDR brachytherapy with single-pulse dose of 0.65 Gy up to 30 Gy (37.10 Gy EQD2, α/β = 3 Gy). For sole brachytherapy, we exclusively used PDR brachytherapy in two sessions with single-pulse dose of 0.65 Gy up to 30 Gy per session. In total, we applied 60 Gy (74.20 Gy EQD2, α/β = 3 Gy). A typical arrangement of needles and the corresponding dose distribution are demonstrated in Fig. 2. For conversion into 2‑Gy single doses, we used an approach similar to that of Lettmaier and collegues [6].

### Follow-up

Patients were scheduled for follow-up appointments every 3 months, during which PSA levels and side effects were recorded. At these visits, patients were asked to complete established EORTC, IIEF, IPSS, and ICIQ questionnaires. These tools were utilized to assess potential toxicity and track the evolution of early and late treatment-related side effects.

## Results

### Patient characteristics

Five patients were identified with previously treated colorectal cancer and disease-related rectal extirpation. Among these, three were diagnosed with high-risk prostate cancer, one with high-intermediate risk prostate cancer, and one had experienced a recurrence after initially being diagnosed with low-risk prostate cancer. Four patients had received prior EBRT of the pelvis during the course of primary treatment for rectal cancer, and one patient was radiotherapy naive. For four patients, pretreatment (neoadjuvant chemoradiation and rectal surgery) was deemed a contraindication for surgical intervention by the multidisciplinary tumor board. One patient declined surgery due to personal preference. For further details, see Table 1.

According to national guidelines, hormonal ablative therapy was initiated prior to or during the course of our treatment when indicated.

### Dosimetric considerations

For brachytherapy we used 10–12 titanium needles with a length of 200 mm. We documented the following median values: V100 94.7% (94.5–98.4%), D2_bladder_ 64.3% (50.9–78.3%), D10_urethra_ 131.05% (123.2–141.2%), and D30_urethra_ 122.45% (116.2%–129.5%). CTV volumes ranged from 20 to 36.06 ccm, with a median volume of 21.67 ccm. Additionally, we reconstructed pretreatment dosing. All dose constraints were met. For further details see Table 2.

**Table 2:** Treatment characteristics

Patient/procedure	1.1	2.1	3.1	3.2	4.1	4.2	5.1
No. catheters	12	11	10	10	4	4	11
HDR/PDR	PDR	HDR	PDR	PDR	PDR	PDR	PDR
Total dose	29.9	18	30	30	30	30	30
Pulse dose/single dose	0.65	9	0.65	0.65	0.65	0.65	0.65
DNR	0.51	0.48	0.40	0.35	0.55	0.568	0.377
V100 (%)	94.52	94.60	94.80	98.40	97.07	93.73	94.92
V150 (%)	57.80	22.44	42.40	40.10	72.08	64.43	38.68
V200 (%)	31.59	–	18.50	15.30	50.26	43.98	16.28
D90 (%)	107.80	111.4	114.30	107.70	119.60	108.10	106.6
D10 urethra (%)	141.20	133.10	123.20	81.05	X^a^	X^a^	125.50
D30 urethra (%)	129.50	122.80	116.20	129.00	X^a^	X^a^	121.00
D2 bladder (%)	70.90	704.65	37.53	33.06	X^a^	X^a^	73.0

*HDR* High Dose Rate, *PDR* Pulse Dose Rate, *DNR* Dose Non-Uniformity Ratio

^a^Patient underwent cystectomy.

### Safety

No serious toxicity grade 2 or higher was documented. In particular, there were no reported side effects like acute bleeding, infection, or organ puncture. Notably, one patient already presented with uro-/colostoma after disease-related extirpation during the earlier course of treatment (2018). Therefore, documentation of urogenital toxicity was only possible in 4/5 patients.

All patients noted a decline in sexual function, which was primarily attributed to the initiation of hormonal ablative therapy, as per current guidelines. IPSS scores remained stable in 2 patients before and after treatment. One patient reported improvement of obstructive symptoms after radiotherapy. A trend was also observed concerning incontinence. There were no substantial differences in the documented incontinence scores according to ICIQ questionnaires. Furthermore, one patient experienced hematuria, which was self-limiting and did not require intervention.

Quality of life according to EORTC did not change after the procedure and was in the upper half of the scale for all patients.

### Efficacy

During the follow-up period up to the date of final analysis, no local recurrence was reported. One patient (patient 3 in Table 1) presented with a singular metastasis after 35 months of follow-up. No local recurrence was documented. In 4/5 patients, PSA values remain undetectable to date. Hormonal therapy was ongoing during the follow-up period of all patients. The nadir was reached within 3 months after initiation of therapy in all patients.

## Discussion

Interstitial brachytherapy as a curative treatment option for prostate cancer is well established in various international guidelines [7–10]. It is recommended not only for low-risk cases, where brachytherapy alone suffices, but also for intermediate- to high-risk cases in combination with external-beam radiation therapy (EBRT), marking it a state-of-the-art therapy with a favorable toxicity profile [11]. This fact is also reflected in numerous international guidelines for the treatment of prostate cancer and the recommendation has remained unchanged for several years [7–10].

Additionally, brachytherapy shows high efficacy in the case of recurrence without metastasis after primary treatment (definitive radiotherapy or radical prostatectomy with postoperative EBRT) [12]. When pretreated with radiotherapy, the challenge in such situations is mainly related to the localization of the recurrent tumors with respect to already applied doses to organs at risk. While salvage surgery or EBRT could be considered, these treatments often carry a significant risk of both early and late toxicity. Given the known dose–response relationship, the use of EBRT is very limited due to already exhausted normal tissue tolerance [13, 14]. In contrast, salvage brachytherapy offers an acceptable toxicity profile with high local control rates and is therefore the treatment of choice [15, 16]. One out of 5 patients in our analysis presented with recurrent PCA. He had received pretreatment with iodine seeds for low-risk prostate cancer. Despite having a primary PCA diagnosis, the other patients (4/5) had mostly also been pretreated with EBRT for rectal cancer, which has to be considered for treatment planning. As a result, primary surgical removal and EBRT were not favored options due to the high risk of serious genitourinary (GU) and gastrointestinal (GI) toxicity [17]. ABS guidelines generally recommend transrectal ultrasound as the imaging method [18, 19]. However, also CT-based planning systems are mentioned, analogous to the planning of several other entities in the area of brachytherapy such as breast cancer or cervical carcinoma [20, 21].

The application of brachytherapy in patients who have undergone rectal extirpation and subsequently develop metachronous prostate cancer has been reported in just a couple of case series to date [22, 23]. Similarly to our approach, Koutrovelis et al. used CT-assisted image guidance for low-dose-rate (LDR) brachytherapy with iodine seeds alone (in patients with mostly intermediate- to low-intermediate-risk prostate cancer). After a median follow-up of 18.6 months, excellent biochemical control was reported, despite one instance of biochemical failure. Importantly, patients did not experience any gastrointestinal morbidity. One patient had a stricture of the distal ureter which requiring stenting [22]. Notably, we found similar toxicity in the same follow-up period without documenting serious toxicity requiring intervention. Jabbari et al. also investigated rectum-extirpated patients with newly diagnosed prostate cancer but used transperineal ultrasonographic image guidance. Excellent local control during the follow-up period was reported. Notably, 2 patients reported late grade 2 genitourinary toxicity which might be explained by insufficient image quality during the procedures [23].

Given the advancements in cancer therapy, the improved prognosis of colorectal cancer, and a noticeable increase in the prostate cancer incidence following rectal cancer, it is likely that the number of patients with these specific clinical features will increase in the future [1]. In this context, the novelty we were able to contribute with our analyses is that we offer an option to patients who are otherwise inaccessible to curative treatment. A recent meta-analysis described 89 patients with metachronic colorectal and prostate cancer of whom 23 received brachytherapy; 9 patients in 2 studies were described as rectally extirpated and receiving interstitial BT [24]. This shows that the scenario described in our analysis is prevalent, but there is a significant lack of data for a standardized treatment approach.

In both of the abovementioned studies [22, 23], brachy-therapy alone was used. In our report, we are able to demonstrate for 2 patients that also a combined approach, normofractionated EBRT and BT, is feasible. Since one patient remained radiotherapy naïve after the earlier rectal cancer treatment, reirradiation was nonproblematic. For another patient, radiographic evidence suggested nodal involvement, which is why we decided to treat the pelvic nodal regions as well. Interestingly, this patient did not report increased GI or GU toxicity during the course of follow-up.

Another novelty we were able to demonstrate is that the suggested method is also feasible for patients with recurrent disease after prostatectomy. To our knowledge, there are no reports of such cases in the literature.

The presented analysis has several limitations. Certainly, the low number of evaluated procedures in this analysis must be considered. In addition, the follow-up period might seem rather short in order to give conclusive results regarding efficacy compared to other image-guided brachytherapy approaches. Furthermore, the presented procedure is highly dependent on the executing brachytherapy expert in terms of experience and technical equipment, thus restricting this treatment option to high-volume institutions with a specialization in interstitial brachytherapy. However, for patients with prostate cancer who have undergone surgery and chemoradiation for rectal cancer and are thus not candidates for ultrasound-guided brachytherapy or radical surgery due to contraindications or personal preference, CT-guided brachytherapy remains a possible curative option. Nevertheless, further data are required to draw conclusions about the efficacy of the proposed method.

In conclusion, our findings suggest that protocol-based CT-guided brachytherapy is a feasible and safe approach for prostate cancer patients who have previously undergone rectal extirpation with or without radiotherapy.
